# Sucrose-Related Transcriptomic and Metabolic Profiles Are Associated with Latex Yield Variation in Three *Hevea brasiliensis* Clones

**DOI:** 10.3390/plants15213442

**Published:** 2026-11-05

**Authors:** Changli Mao, Xia Zeng, Tian Yang, Chenwanli Li, Cheng Zheng, Junming Ai, Xiaodong Liu, Jing Zhang, Xiaoping Zhu, Haoqi Shi, Yu Wu, Jin Liu

**Affiliations:** 1 Yunnan Technology Innovation Center of Natural Rubber, Yunnan Key Laboratory of Sustainable Utilization Research on Rubber Tree, Yunnan Institute of Tropical Crops, Xishuangbanna 666100, China; maokangwan@163.com (C.M.); z_cheng.93@foxmail.com (C.Z.);; 2 State Key Laboratory of Tropical Crop Breeding, Sanya 572024, China

**Keywords:** *Hevea brasiliensis*, rubber yield, RNA-Seq, sucrose metabolism, multi-omics

## Abstract

Natural rubber from *Hevea brasiliensis* serves as an irreplaceable industrial raw material. Whether rubber-yield potential is constrained by sucrose-sink sucrose partitioning remains incompletely understood. Here, we integrated transcriptomics and targeted sugar metabolomics across leaves, bark and latex from three *H. brasiliensis* clones with contrasting yields (designated H, M, and L). Transcriptomic profiling revealed tissue-specific functional differentiation: leaves were enriched in photosynthetic carbon fixation, bark in substance transport, and latex in defence and regeneration. The H clone did not show enhanced transcriptional enrichment of photosynthetic pathways relative to the L clone. Instead, it displayed a higher transcript abundance of invertase and sucrose synthase genes alongside elevated hexose levels, pointing to putative genotypic differences in sucrose hydrolysis and hexose turnover. Metabolomic profiling revealed greater sucrose accumulation in leaves of the L clone, accompanied by relatively low sucrose concentrations in latex. WGCNA analysis identified the MEpink module as strongly correlated with leaf sucrose content. This module is enriched in thiamine and cyanoamino acid metabolism and contains candidate transcription factors *WRKY40*, *MYC2* and *NAC002*. Our results suggest that yield variation among these clones correlates with distinct sucrose distribution and utilization profiles. These multi-omics signatures provide a molecular resource to support high-yield rubber tree breeding.

## 1. Introduction

Natural rubber (cis-1,4-polyisoprene) possesses unique elasticity, abrasion resistance, and tear strength, granting it an irreplaceable strategic role in critical sectors such as aerospace, transportation, medical protection, and defense [1,2]. Relative to synthetic rubber, natural rubber has superior dynamic mechanical properties, sustaining steadily rising global market demand [3]. Currently, the Brazilian rubber tree (*Hevea brasiliensis*) remains nearly the only commercial source of natural rubber, with its cultivation areas mainly concentrated in tropical regions of Southeast Asia, Africa, and South America [4,5,6]. Despite its economic importance, rubber production faces multiple constraints, including diseases, limited genetic variation, and restricted climatic adaptation, highlighting the need to explore alternative sources and improve existing cultivars. Nevertheless, as a perennial tropical tree species, *H. brasiliensis* features a long breeding cycle of 30–40 years. Compounded by climate variability and frequent disease outbreaks in recent decades, the global supply demand imbalance for natural rubber has intensified [7]. Accordingly, in-depth elucidation of the molecular regulatory mechanisms underlying rubber yield formation in the rubber tree and the breeding of new high-yielding and high-quality varieties hold important theoretical value and practical significance for ensuring the security and stability of the global natural rubber supply chain.

Sucrose is integral to plant physiology, acting as a primary transport form of sugar translocated from the leaves via the phloem to other parts of the plant, such as stems, fruits, and roots, which function as storage tissue roots (sink tissues) [8,9,10,11]. Starch is synthesized and stored in the leaf chloroplasts and amyloplasts of the parenchyma tissues. Fruits of certain plants also serve as reservoirs for starch and sucrose. These carbohydrates are crucial during the initial growth stages and bud development because they are the sole carbon sources during these periods [8,9,12,13]. Additionally, these carbohydrates are pivotal in responses against environmental stresses like drought or pest attacks [14,15], ensuring plant longevity and health [16,17,18]. Rubber trees are especially prone to physiological stress due to the intense tapping during latex harvesting. The latex contains 40% dry rubber particles, and its extraction generates an artificial carbohydrate sink, creating natural carbohydrate storage needed for maintenance, growth, and reproduction [19,20]. Research has shown that tapping can increase the starch reserves within the trunk of rubber trees, albeit at the expense of radial growth, compared to non-tapped trees. In these trees, starch serves as a crucial long-term storage medium in the wood and maintains the equilibrium of carbohydrate levels within the bark. Soluble sugars, especially sucrose, act as intermediaries for latex production in the bark and starch synthesis in the wood [18,21].

Sucrose concentration is directly linked to latex production, with studies demonstrating a significant correlation between the two [22,23,24]. The expression of sucrose transporter *HbSUT3*, which mediates sucrose movement into laticifers, increases in response to ethylene application and the mechanical act of tapping, both of which enhance latex output [25,26]. Alkaline/neutral invertase cleaves sucrose into hexose sugars, which are then exploited in subsequent latex production [22,27]. Additionally, the invertase enzyme, particularly the alkaline/neutral invertase, catalyzes the breakdown of sucrose into simpler sugars that are then utilized in latex production, with its activity being positively correlated with latex yield [28]. Treating Hevea bark with Ethrel, an ethylene-releasing compound, significantly increases the transtonoplast ΔpH within Hevea laticifers, serving as one of the main mechanisms by which ethylene boosts latex yield [29,30]. These findings indicate that latex production is governed by an intricate network involving gene regulation, multi-enzyme reactions, and complex physio-biochemical processes.

Latex yield of *H. brasiliensis* is a complex quantitative trait controlled by multiple factors. Genetically, yield variation among clones is tightly linked to structural traits such as laticifer number and dry rubber content [31]. The yield increase in cultivated varieties compared with wild relatives is largely attributable to the increase in the number of laticifer rings [32]. Environmentally, temperature and precipitation exert substantial impacts on yield. Areas with low temperature fluctuation and adequate rainfall are characteristic of high-yielding rubber producing regions [32,33,34,35]. Furthermore, tapping and chemical stimulation represent important anthropogenic regulators [36,37,38]. The application of stimulants such as ethylene and jasmonic acid has demonstrated that ethylene stimulation promotes latex generation by regulating multiple biological processes, including glycometabolism, energy metabolism, turgor pressure, pH value, and post-translational modifications [38,39,40,41]. In contrast, jasmonic acid improves rubber productivity via the induction of laticifer differentiation [36]. Among these regulators, sucrose serves as the primary direct carbon precursor for rubber biosynthesis. Previous work has reported that the expression of the latex-localized sucrose transporter gene *HbSUT3* is strongly positively correlated with rubber yield, demonstrating that efficient sucrose transport constitutes a key limiting factor for high yield [25].

Metabolites play a crucial role in plant metabolism, notably affecting plant biomass and structure [42,43,44]. Metabolomics has become a cutting-edge technology, enabling detailed metabolite profiling across various organisms, including microbes, plants, and animals [45,46]. This technology allows for the rapid and accurate analysis of a wide spectrum of metabolites from a single extract, providing a holistic view of cellular metabolites and thus capturing the complete physiological state of a cell. The advanced metabolomics techniques facilitate the study of mutants and genetically modified lines to unravel complex metabolic networks and identify key genes responsible for specific metabolic traits [47,48]. Furthermore, metabolomics aids in elucidating gene functions, their impact on metabolic pathways, and their intricate regulation and interactions within these pathways, which are often not discernible through traditional methods like microarray analyses [49,50,51]. Through transcriptome analysis, various genetic regulators have been identified, including M-type MADS, MYB (v-myb avian myeloblastosis viral oncogene homolog), MYB-related, and NAC (NAM, ATAF1/ATAF2, and CUC2) families, which are implicated in the regulatory processes of the rubber tree [52].

While prior research has explored the mechanisms underlying rubber yield formation, most studies have focused on a single tissue, (predominantly latex, or a single omics layer. Systematic characterization of coordinated sugar metabolism across leaves, bark, and latex remains limited. To address these gaps, we selected three genetically distinct rubber tree clones (designated H, M, and L) with divergent field measured latex yields. By integrating transcriptomics and targeted sugar metabolomics, together with weighted gene co-expression network analysis (WGCNA), we systematically examined tissue-specific sucrose distribution and its underlying molecular regulatory network. This work aims to provide new mechanistic insights into yield variation in rubber trees and lay a theoretical foundation for high-yield breeding and the development of laticifer-based bioreactors.

## 2. Results

### 2.1. Comparative Transcriptomic Analysis of Rubber Tree Clones with Contrasting Yields

To uncover the molecular basis of rubber tree yield formation, transcriptome sequencing was performed on the bark, leaves, and latex of clones H, M and L, which differed in measured latex yield. In total, 27 cDNA libraries were constructed. Clean reads accounted for 82–97% of raw reads. Clean reads were mapped against the *H. brasiliensis* reference genome, and gene expression was quantified in nine comparison groups: L-latex vs. M-latex, L-latex vs. H-latex, M-latex vs. H-latex, L-bark vs. M-bark, L-bark vs. H-bark, M-bark vs. H-bark, L-leaf vs. M-leaf, L-leaf vs. H-leaf, and M-leaf vs. H-leaf. Sample names and codes are listed in Supplementary Table S1.

**Figure 1:**
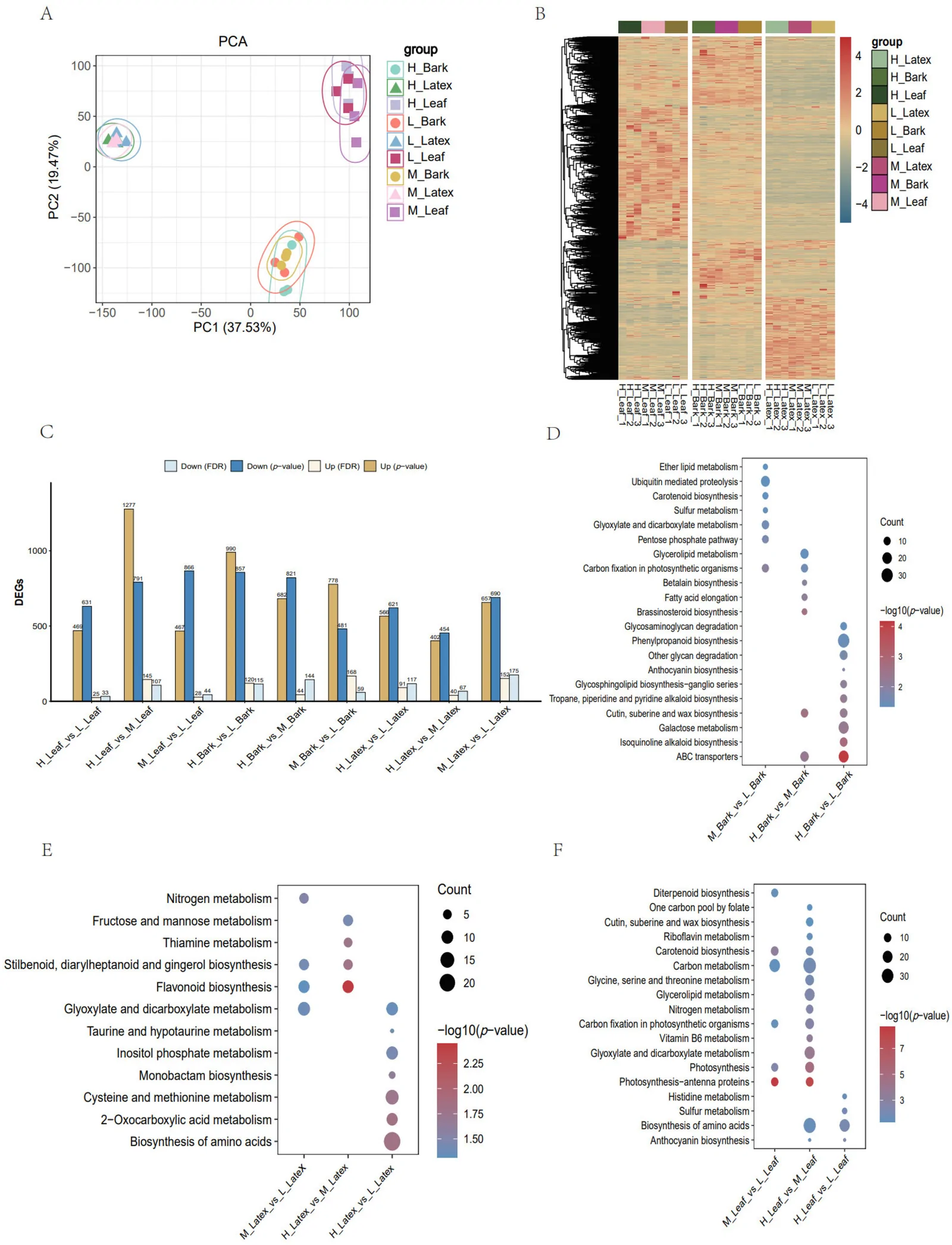
Transcriptomic analysis of rubber tree clones with different latex yields. (**A**) Principal component analysis (PCA) plot based on transcriptome data, showing the distribution of leaf, bark, and latex samples from H, M, and L clones. (**B**) Cluster heatmap displaying the overall gene expression patterns across all samples. (**C**) Bar plot showing counts of up- and downregulated genes for each tissue and pairwise clone comparison. Light bars represent FDR-significant DEGs (BH-adjusted *p* < 0.05, |log_2_(fold change)| >1). Dark bars denote exploratory candidate genes derived from uncorrected nominal *p* < 0.05 and |log_2_(fold change)| >1; this exploratory set was used for KEGG enrichment and WGCNA intersection and was not corrected for multiple-testing bias. (**D**–**F**) KEGG pathway enrichment analysis plots showing significantly enriched pathways of candidate genes in leaves (**D**), bark (**E**), and latex (**F**), respectively. For KEGG enrichment analysis, the input gene sets were defined using *p* < 0.05 and |log_2_(fold change)| > 1.

Principal component analysis indicated a clear separation among samples from different tissues (Figure 1A). Cluster heatmap analysis further demonstrated that leaves exhibited the highest overall transcriptional activity (Figure 1B). To ensure statistical rigor, a strict threshold of BH-adjusted *p* < 0.05 and |log_2_(fold change)| > 1 was first applied, identifying a core set of 1674 statistically confirmed DEGs (Supplementary Data S1). For hypothesis-generating exploratory downstream analyses, including KEGG enrichment and WGCNA module-DEG intersection, we additionally applied a nominal uncorrected threshold of raw *p* < 0.05 and |log_2_(fold change)| > 1 to obtain 12,500 exploratory candidate genes. This exploratory gene set was not corrected for multiple testing and should be interpreted with caution. Within this exploratory set, 6288 genes were upregulated and 6212 genes were downregulated. The distribution of these candidate genes varied across tissues and comparisons, with the highest number in leaves and the lowest in latex (Figure 1C, Supplementary Data S1). This suggests that compared to leaves, which are highly susceptible to environmental influences, latex may harbor a more conserved core regulatory module for yield.

KEGG enrichment analysis of our exploratory candidate gene set revealed tissue-specific distribution patterns of pathways potentially linked to latex yield (Figure 1D–F, Supplementary Data S2). In leaves, these exploratory candidates were significantly enriched in photosynthesis, carbon metabolism, and glycolysis/gluconeogenesis. These enrichment patterns were observed in both H vs. M and M vs. L comparisons, and they provide exploratory evidence that leaf photosynthetic carbon assimilation may contribute to yield variation. In bark, enriched pathways primarily involved protein degradation and transport, secondary metabolite biosynthesis, and cell wall metabolism, exhibiting the strongest signals in comparisons between the H and L clones. This reflects the regulatory potential of bark in material metabolism and vascular development. In latex, these exploratory candidate genes were mainly enriched in flavonoid biosynthesis, amino acid metabolism, and sulfur metabolism, pathways that may be linked to defense response and latex regeneration.

Overall, the transcriptomic profiles point to a putative tissue-coordinated pattern underlying latex yield variation: leaves show transcriptional features related to photosynthetic carbon assimilation that may shape source properties, bark exhibits signatures associated with substance transport and metabolism potentially linked to flow dynamics, and latex displays expression profiles consistent with defence and regeneration processes that may support sink function. These transcriptomic characteristics provide an exploratory basis for the further investigation of key metabolic pathways potentially linked to yield variation.

### 2.2. Targeted Glycomics Reveals Differences in Sugar Profiles Across Tissues and Clones

**Figure 2:**
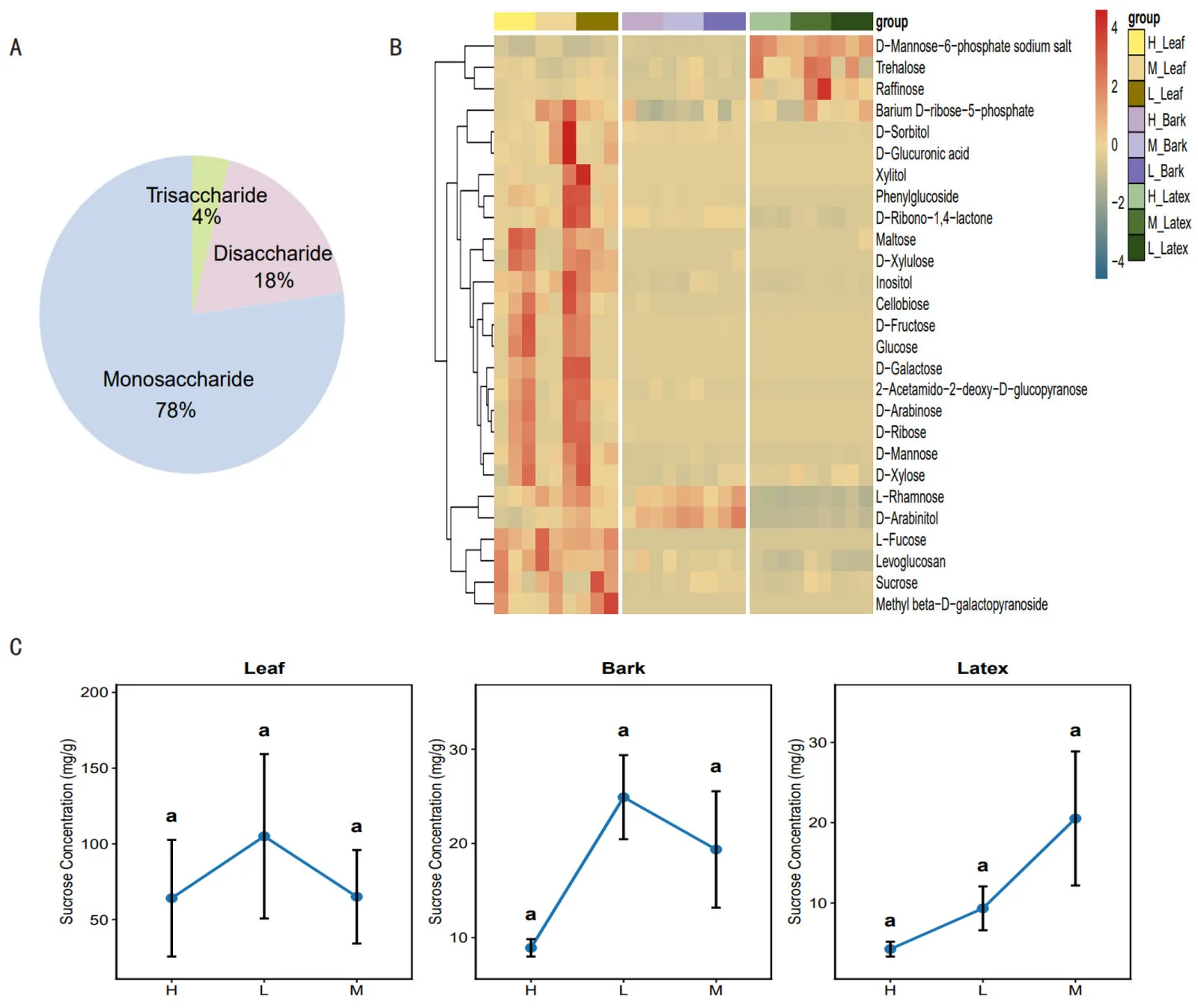
Targeted sugar metabolomics analysis. (**A**) Pie chart showing the classification of detected sugar metabolites. (**B**) Heatmap of all sugar metabolites, displaying the abundance distribution of sugar metabolites in samples from different tissues (leaf, bark, latex) and different clones. (**C**) Line chart of sucrose content (mg/g) across different tissues and three clones. Sucrose was quantified by external standard calibration using the internal standard method; the measured peak area ratio was substituted into the corresponding linear equation to calculate the metabolite content. Error bars represent standard error of the mean (SEM) from three biological replicates (*n* = 3). Letters denote results from one-way ANOVA followed by Tukey’s HSD test (*p* < 0.05); identical letters indicate no significant difference between comparisons.

Sugars such as sucrose serve as direct carbon sources and energy suppliers for natural rubber biosynthesis, so their distribution and accumulation across tissues critically influence latex yield. To further dissect differences in sugar metabolism among rubber tree clones with varying latex yield, we performed a GC-MS based widely targeted metabolomic analysis on leaf, bark, and latex samples from the H, M, and L clones (Supplementary Data S3a). The coefficient of variation (CV) cumulative distribution of metabolite peaks from QC samples was assessed, where over 80% of peaks had CV values less than 0.3, confirming satisfactory repeatability of the analytical platform (Supplementary Figure S1). In total, 27 classes of carbohydrate metabolites were detected across all tissues (Supplementary Data S3b), and 24 DAMs were detected across all comparisons (Supplementary Data S3c). Calibration equations, coefficients of determination (R^2^), LOD, and LOQ for all quantified sugar metabolites are provided in Supplementary Data S4; all curves showed R^2^ > 0.99. Detected sugar metabolites were classified into three categories, with monosaccharides accounting for the highest proportion (78%), while disaccharides and trisaccharides comprised 18% and 4%, respectively (Figure 2A). Hierarchical clustering analysis of all sugar metabolites revealed distinct accumulation patterns across tissues and three clones (Figure 2B). Specifically, leaf samples displayed a high abundance of multiple sugars, whereas bark and latex generally contained lower levels of sugars.

We further characterized sucrose concentrations across the leaf, bark and latex tissues of clones H, M and L. Within each tissue, pairwise comparisons after multiple-testing correction revealed no statistically significant differences in sucrose concentration among the three clones. Nevertheless, we observed consistent numerical trends: the L clone displayed the highest numerical sucrose level in leaves, the L clone showed the highest numerical sucrose value in bark, and the M clone had the highest numerical sucrose concentration in latex (Figure 2C). Collectively, these data reveal clone-associated numerical trends in sucrose partitioning across source and sink tissues, although these sucrose differences did not reach statistical significance.

### 2.3. WGCNA Identifies Co-Expression Modules Associated with Sugar Metabolism Regulation

**Figure 3:**
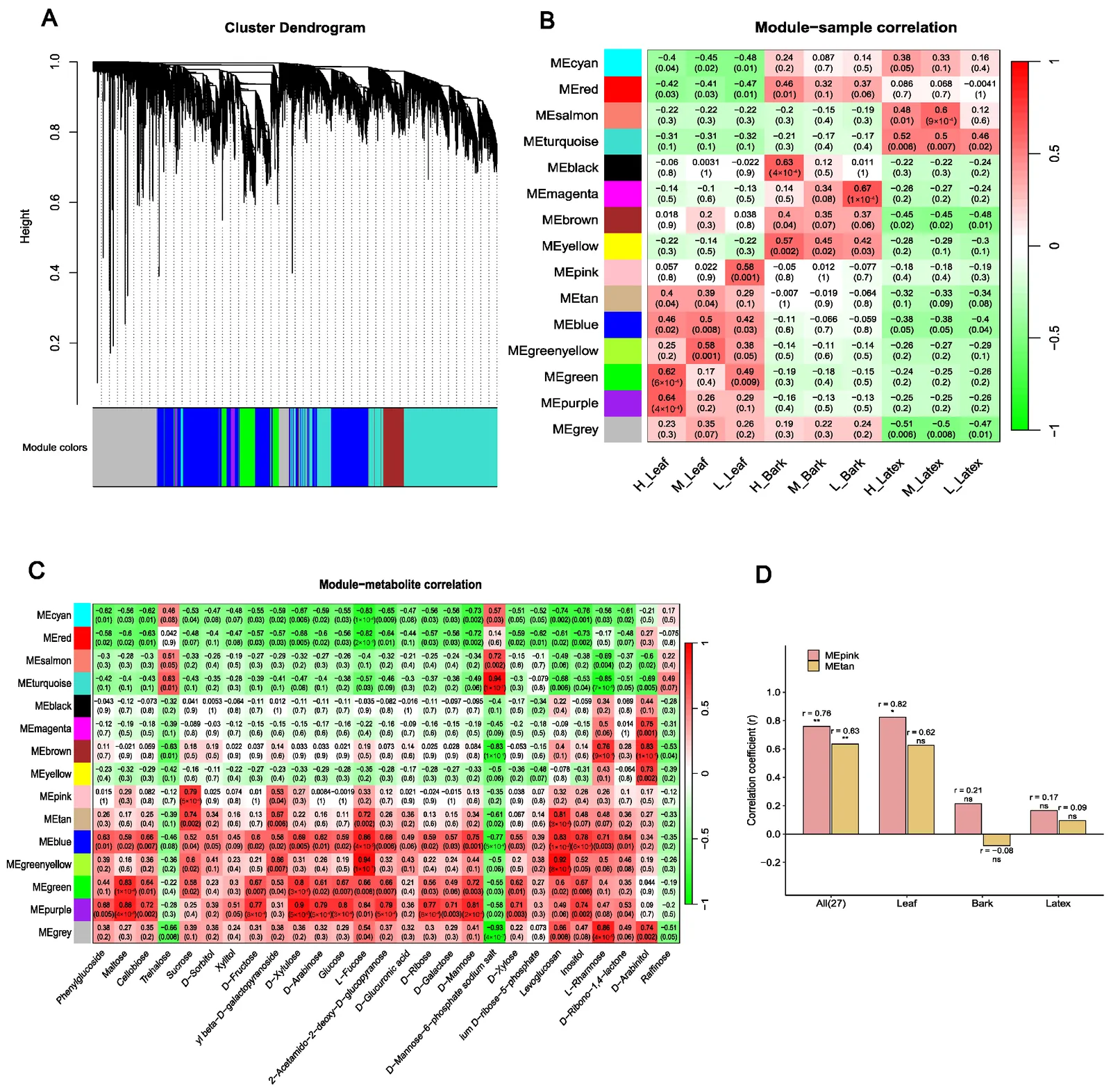
WGCNA analysis of transcriptome data. (**A**) Hierarchical clustering dendrogram showing the division of genes into co-expression modules. (**B**) Module–sample correlation heatmap, displaying the correlations between each module and different samples (leaf, bark, latex, including H, M, L clones). (**C**) Module–metabolite correlation heatmap, displaying the correlations between each module and sugar metabolites. (**D**) Tissue-stratified correlation analysis between module eigengenes (MEpink and MEtan) and sucrose content. Pearson correlations were computed across all 27 samples (All) and within each tissue separately (Leaf, Bark, Latex; *n* = 9 per tissue). Bars represent correlation coefficients (r); asterisks indicate significance levels based on Benjamini–Hochberg adjusted *p*-values (* 0.01 < *p* < 0.05, ** *p* < 0.01; ns, not significant).

To explore the co-expression network of genes regulating tissue development and sugar metabolism in rubber trees, a weighted gene co-expression network analysis (WGCNA) was performed. As shown in Figure 3A, a total of 15 co-expression modules were identified through hierarchical clustering analysis, with each module distinguished by a distinct color. In WGCNA, each module is represented by its module eigengene (ME), defined as the first principal component of the module’s expression matrix. These color identifiers (e.g., MEpink, MEtan, MEblue) are arbitrary identifiers assigned by the WGCNA algorithm and do not imply biological function. Biological relevance of these modules was assessed by correlating MEs with tissue types, clones, and sugar metabolites (Figure 3B–D). Module–sample correlation analysis revealed significant tissue specificity (Figure 3B). Among them, MEpink, MEtan, MEblue, MEgreenyellow, MEgreen, and MEpurple were positively correlated with leaf samples but negatively correlated with bark and latex. Notably, MEpink was positively correlated with leaf tissue from the H clone in global analysis (Figure 3B). Module–metabolite association analysis further showed that several modules were significantly positively correlated with key sugar metabolites (Figure 3C).

To evaluate module and metabolite associations and potential confounding by tissue identity, we correlated MEs with sucrose. Across all 27 samples, MEpink and MEtan were positively correlated with sucrose content (*r* = 0.76, and *r* = 0.63, respectively; Figure 3D). Because both modules are leaf-enriched and sucrose is higher in leaves, these global correlations may be confounded by tissue. We therefore performed tissue-stratified and partial correlation analyses. Within leaf, MEpink remained significantly correlated with sucrose (*r* = 0.82, *p* = 0.0064, BH-adjusted *p* = 0.038), whereas correlations in bark and latex were not significant. MEtan showed no significant within-tissue correlation, although the leaf correlation was moderate (*r* = 0.62, *p* = 0.072). After controlling for tissues and clones, both MEpink and MEtan remained positively associated with sucrose (MEpink: β = 4.33, partial *r* = 0.76, *p* = 2.7 × 10^−5^; MEtan: β = 8.59, partial *r* = 0.59, *p* = 0.003) (Figure 3D, Supplementary Table S2). These results suggest that the MEpink–sucrose association is robust within leaf tissue, while the MEtan–sucrose association also persists after adjustment but should be interpreted cautiously given the non-significant within-tissue correlations. The complete WGCNA outputs, including module memberships, module eigengenes, and correlation matrices, are provided in Supplementary Data S6.

### 2.4. Functional and Expression Analysis of Core Modules

**Figure 4:**
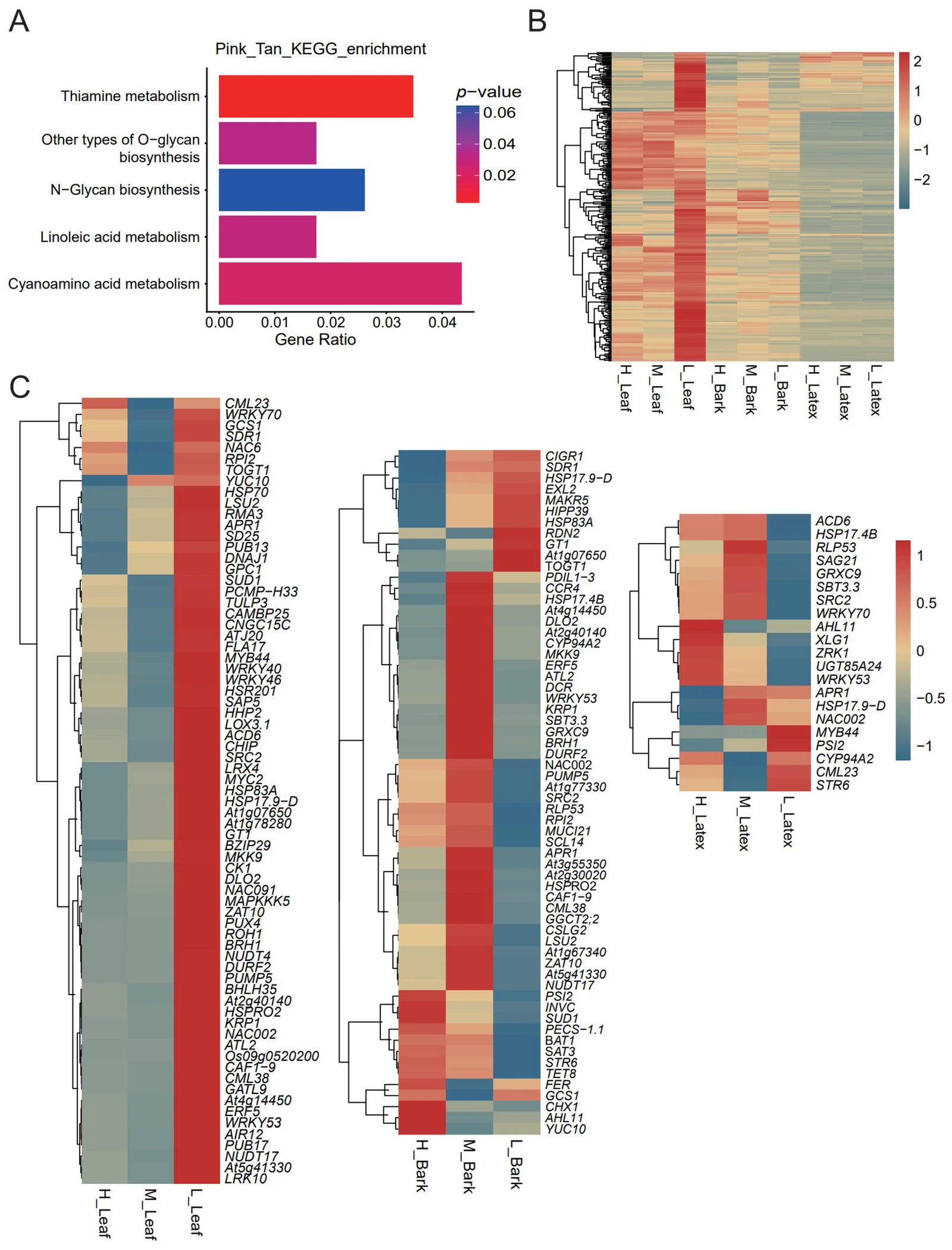
Functional and expression analysis of the MEpink and MEtan modules. (**A**) KEGG pathway enrichment analysis showing the significantly enriched pathways of candidate genes in the MEpink and MEtan modules. (**B**) Expression heatmap showing the abundance distribution of MEpink and MEtan module candidate genes across different tissues (leaf, bark, latex) and different clones. (**C**) Expression heatmap of overlapping genes showing the abundance of genes intersecting between differentially expressed genes from pairwise comparisons among clones H, M, and L in each tissue and the MEpink and MEtan modules across different tissues.

To elucidate the biological functions of the modules associated with sucrose metabolism, we focused on MEpink, which showed a robust leaf-associated correlation with sucrose, and also examined MEtan as a tissue-influenced module. KEGG pathway enrichment analysis was performed on the genes of the two modules. The results showed that these genes were significantly enriched in thiamine metabolism and cyanoamino acid metabolism pathways (Figure 4A), suggesting that these pathways may participate in rubber yield formation by influencing the balance of carbon and nitrogen metabolism. Further analysis of the expression patterns of module genes across different tissues and clones revealed that, as shown in the heatmap, these genes exhibited high expression levels overall in leaves, with most genes showing slightly higher expression intensity in L leaves compared with H and M leaves (Figure 4B).

To screen candidate genes associated with yield differences among the studied clones, we identified overlapping genes between candidate genes from pairwise comparisons among clones in each tissue and genes belonging to MEpink and MEtan. In leaves, 71 overlapping genes were identified, including multiple candidate transcription factors (e.g., *WRKY40*, *WRKY46*, *WRKY53*, *MYB44*, *MYC2*, *NAC002*) and stress-responsive genes (e.g., *HSP70*, *HSP83A*, *LOX3.1*), which were generally highly expressed in L leaves (Figure 4C). In bark, 62 overlapping genes were identified, most of which showed higher expression in M bark, whereas genes such as *PSI2*, *INVC*, and *BAT1* exhibited higher expression in H bark. In latex, 21 overlapping genes were identified, including candidate transcription factors such as *WRKY53*, *MYB44*, and *NAC002*, which showed higher expression in H and M latex.

Collectively, these results suggest that MEpink is associated with sucrose content and expression differences between the studied genotypes.

### 2.5. Integrative Multi-Omics Analysis of the Sucrose Metabolic Pathway

**Figure 5:**
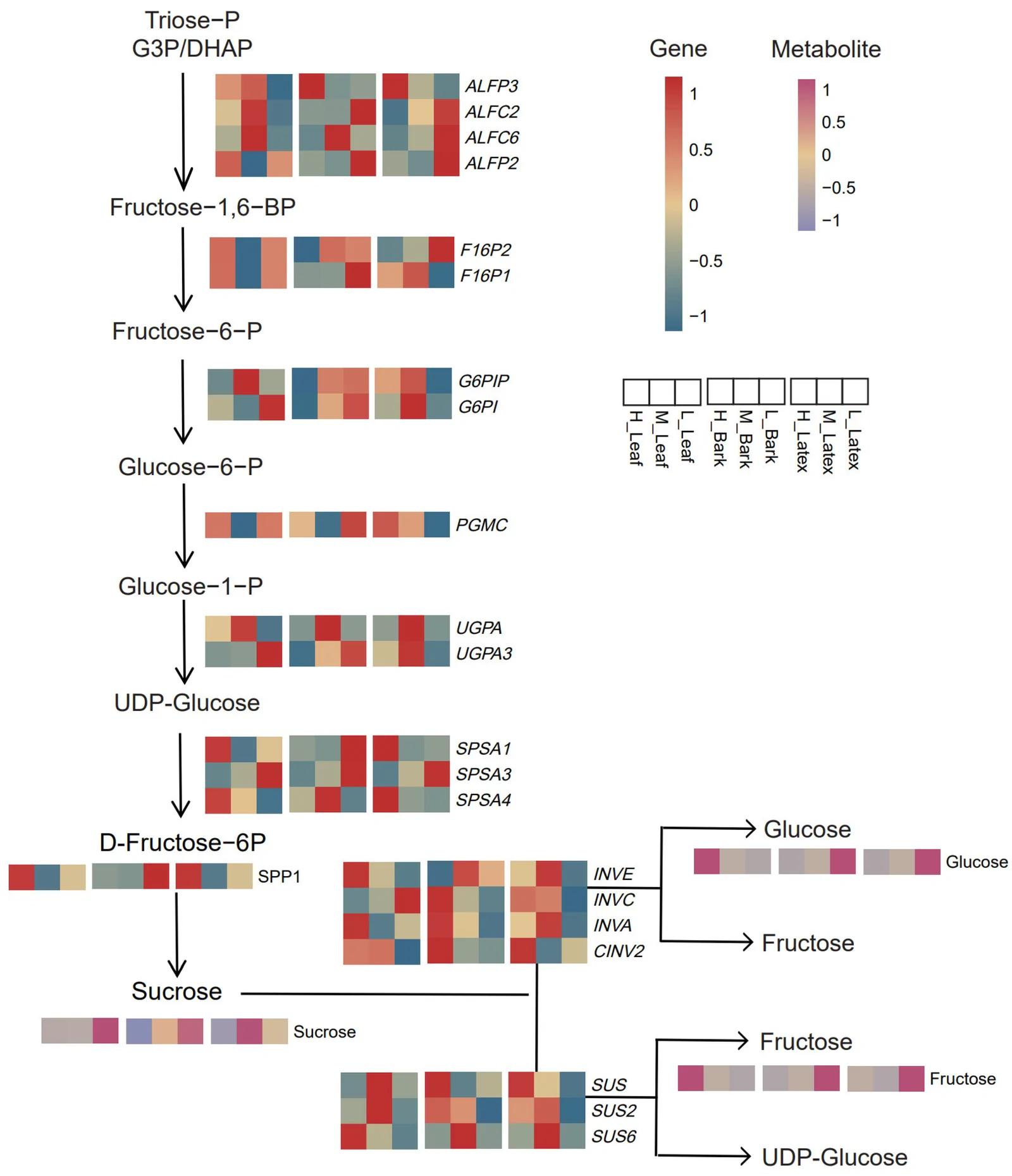
Integrative multi-omics analysis of the sucrose metabolic pathway.

To further characterize the dynamic changes of the sucrose metabolic pathway across different tissues and clones in rubber trees, we integrated transcriptomic and metabolomic data to systematically analyze the expression of key genes and the accumulation of intermediate metabolites involved in the sucrose metabolic pathway (Figure 5). At the gene expression level, aldolase-encoding genes (*ALFP3*, *ALFC2*, *ALFC6*, *ALFP2*), which participate in the conversion of triose phosphates to fructose-1,6-bisphosphate, showed higher expression in H and M leaves, and also exhibited high expression in L latex. Genes encoding sucrose phosphate synthase and sucrose phosphate phosphatase (*SPP1*, *SPSA1*, *SPSA3*, *SPSA4*) were highly expressed in H leaves and H latex while showing elevated expression in L bark. Notably, invertase-encoding genes (*INVE*, *INVC*, *INVA*, *CINV2*) and sucrose synthase-encoding genes (*SUS*, *SUS2*, *SUS6*) were highly expressed across the leaf, bark, and latex tissues in H and M clones. These expression signatures suggest that these genes may act synergistically in sucrose breakdown and downstream hexose processing across tissues, potentially supplying carbon skeletons and energy for rubber biosynthesis.

At the metabolite level, the L clone showed numerically higher sucrose concentrations in leaves and bark, while the M clone exhibited numerically higher sucrose levels in latex, although these differences did not reach statistical significance. Glucose and fructose were more abundant in H leaves, and were also elevated in L bark and L latex. Notably, the presence of accumulated glucose and fructose in L bark and latex, instead of their depletion, provides a suggestive pattern: the L clone may not suffer from limited hexose availability, but could exhibit altered downstream hexose processing. This pattern aligns with the observed gene expression trends and is compatible with the hypothesis of divergent sucrose distribution and utilization. Specifically, the L clone may have modified hexose processing, while H and M clones show signatures of enhanced sucrose breakdown and hexose turnover. However, this hypothesis requires further validation through tracer assays, phloem flux measurement, or functional transporter studies.

In summary, coordinated genotypic differences in sucrose-metabolism pathway profiles were observed across tissues of the three clones. Clone H exhibited an elevated expression of genes involved in sucrose synthesis and catabolism; this expression pattern provides signatures suggestive of enhanced sucrose processing that may supply carbon precursors for latex regeneration. Clone L showed numerically elevated glucose and fructose levels in bark and latex, consistent with potential perturbations in downstream hexose turnover. Our multi-omics data reveal a correlation between genotypic yield variation and distinct sucrose distribution and utilization signatures, yet causal mechanisms remain to be experimentally verified.

### 2.6. Quantitative Real-Time PCR (qRT-PCR) Validation

**Figure 6:**
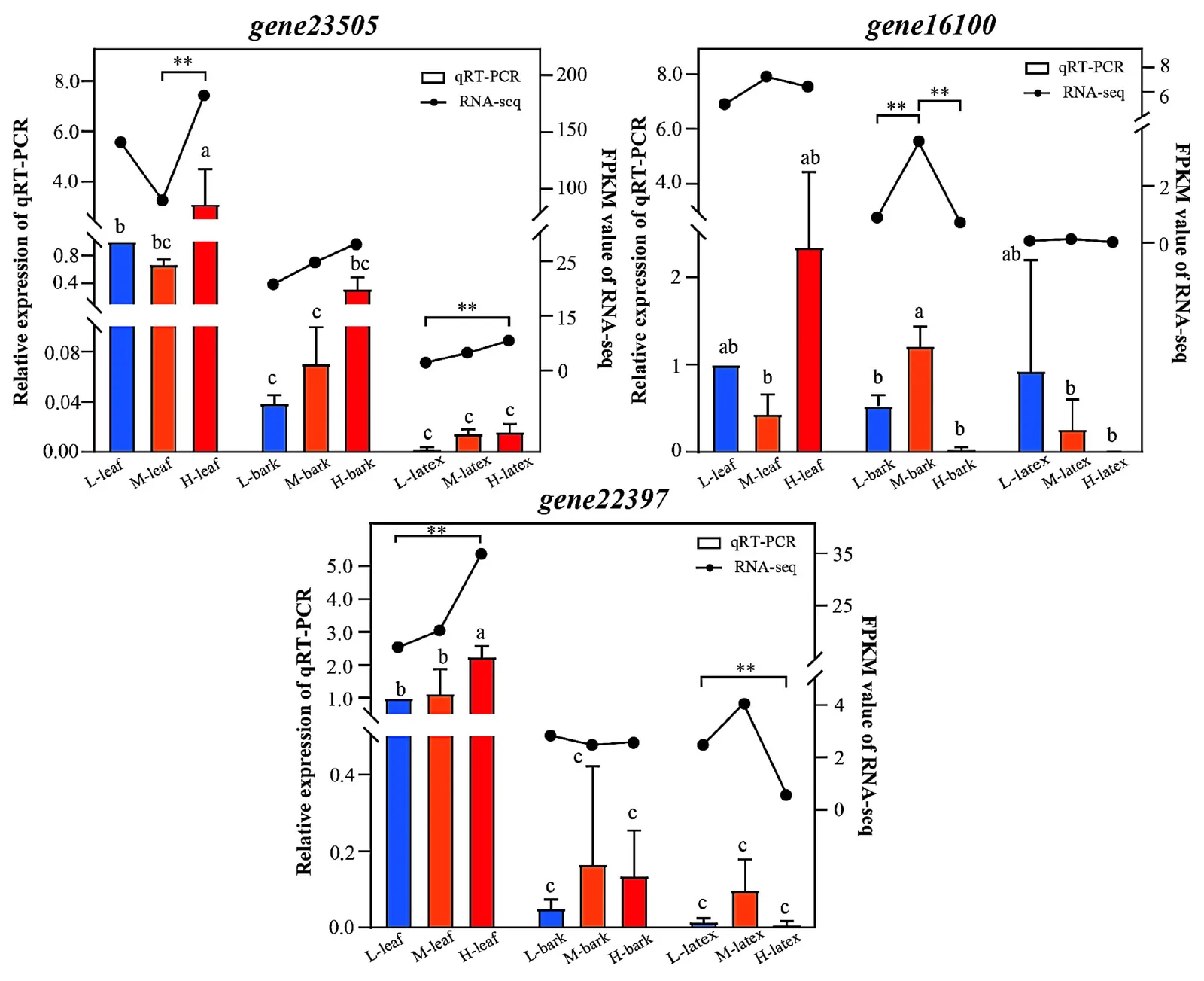
qRT-PCR validation of selected sucrose metabolism related genes (bars denote relative expression levels from qRT-PCR, and black lines show the FPKM values of RNA-Seq. Data are presented as mean ± standard deviation (SD) of three independent biological replicates (*n* = 3), with three technical replicates averaged for each biological sample. Different lowercase letters indicate significant differences among qRT-PCR samples; ** represents an extremely significant difference at *p* < 0.01).

Three key genes for sugar metabolism were selected for qRT-PCR validation. All genes exhibited tissue-specific expression, with the highest transcript abundance in leaves, intermediate in bark, and the lowest in latex. Consistent with the RNA-Seq data, gene23505 and gene22397 showed significant expression differences in leaves, while such differences of gene16100 were detected in bark. Although no significant difference was observed in latex due to low gene abundance, comparable expression trends were retained in two datasets. On the whole, the qRT-PCR results were concordant with the transcriptome data, verifying the reliability of the RNA-Seq results (Figure 6, Supplementary Data S5).

## 3. Discussion

Using integrated transcriptomic and targeted sugar metabolomic analyses, this study revealed the differences in sugar metabolism among the three studied clones with contrasting yields. These findings suggest that the observed sugar profiles and gene expression patterns are compatible with the hypothesis of differences in sucrose distribution and utilization, which warrants future confirmation through tracer experiments, phloem flux measurement, or functional studies of candidate transporters.

Transcriptomic profiling revealed clear functional differentiation across rubber tree tissues: leaves were enriched in photosynthetic carbon fixation pathways, bark in substance transport pathways, and latex in defense and regeneration pathways. These results are consistent with established findings on tissue-specific gene expression in rubber trees, wherein genes related to photosynthesis and carbon fixation are predominantly active in leaves [53], the bark serves as a critical relay station for sucrose transport [54], and latex exhibits intense defense and stress responses concurrent with rubber regeneration [55,56]. Notably, the H clone did not exhibit higher enrichment levels in photosynthetic pathways compared with the L clone. This indicates that the yield differences observed among these specific genotypes are associated with distinct carbon allocation patterns and gene expression profiles. This observation echoes long-standing concepts of source-sink regulation in crop systems, in which source-organ photosynthesis and post-photosynthetic carbon partitioning collectively shape yield performance [57,58,59]. Importantly, photosynthetic gas-exchange parameters and phloem carbon flux were not directly quantified in our experiment; hence we cannot formally evaluate their relative contributions to the observed yield differences, and related interpretations remain hypothetical.

Although absolute sucrose concentrations among the three clones did not reach statistical significance (likely due to substantial biological variance and limited sample size, Figure 2C), a distinct numerical trend was observed: the L clone consistently showed numerically higher sucrose levels in leaves compared with the H and M clones. This trend is consistent with the leaf sucrose retention hypothesis, which remains tentative given the lack of statistical significance. More importantly, the integrated multi-omics data revealed a coordinated trend: the L clone showed higher leaf sucrose levels but relatively lower expression of sucrose-cleaving enzymes, whereas the H and M clones exhibited a higher expression of invertases (*INVE*, *INVC*, *INVA*, *CINV2*) and sucrose synthases (*SUS*, *SUS2*, *SUS6*) alongside lower leaf sucrose levels and higher hexose accumulation. This coordinated trend between metabolites and transcripts is consistent with the hypothesis that the H clone may show transcriptional and metabolic signatures of enhanced sucrose hydrolysis to hexoses, which could supply carbon precursors for rubber biosynthesis [60], whereas the L clone may exhibit altered downstream sucrose processing. Furthermore, invertases may serve as potential molecular valves shaping carbon allocation, and their expression levels have been reported to correlate with rubber yield [28]. Nevertheless, because carbon flux and phloem transport were not directly quantified in our work, these observations remain exploratory, and causal relationships await future functional validation.

Previous studies have identified key sucrose transporters and metabolic enzymes in *H. brasiliensis*. *HbSUT3* plays an active role in sucrose loading to laticifers and rubber productivity [41], while *HbSUT5* regulates intracellular sucrose transport in bark and laticifers [25]. *HbNIN2*, a cytosolic alkaline/neutral invertase, is responsible for sucrose catabolism in rubber-producing laticifers [61], and the sucrose synthase gene family is involved in stress response and sucrose utilization in laticifers [62]. Our finding that *INV* and *SUS* genes are upregulated in the H and M clones is consistent with these reports and extends them by showing coordinated regulation across leaves, bark, and latex. In contrast, the L clone may exhibit altered patterns in phloem loading or sucrose unloading, which is consistent with the non-significant numerical trend toward higher sucrose levels in its source organs. Future studies should quantify the expression and activity of *HbSUT3*, *HbSUT5*, *HbNIN2*, and *HbSUS* family members in the same experimental system.

WGCNA identified two co-expression modules (MEpink and MEtan) that were strongly correlated with sucrose content across all 27 samples (r = 0.76 and 0.63, respectively). To control for the confounding effect of tissue identity, we performed both tissue-stratified and partial correlation analyses (Figure 3D and Table S2). The partial correlation controlling for tissue and clone confirmed that both MEpink (partial r = 0.76, *p* = 2.7 × 10^−5^) and MEtan (partial r = 0.59, *p* = 0.003) retained significant associations with sucrose after removing tissue and clone effects. In contrast, the tissue-stratified analysis showed that only MEpink remained significant within leaf tissue (r = 0.82, *p* = 0.0064, BH-adjusted *p* = 0.038), whereas MEtan showed a moderate but non-significant within-leaf correlation (r = 0.62, *p* = 0.072), likely reflecting the limited statistical power of the within-tissue analysis (*n* = 9). Taken together, these results suggest that MEpink represents a robust, leaf-associated module strongly correlated with sucrose, whereas MEtan exhibits a weaker and more tissue-influenced association.

Genes within both modules were predominantly expressed in leaves, with higher expression in L leaves. Because sucrose is predominantly synthesized in leaves and accumulated to markedly higher levels in L leaves, these modules are associated with leaf-specific variation in sucrose metabolism. Notably, several candidate transcription factors (TFs) within these modules—*WRKY40*, *WRKY46*, *MYC2*, and *NAC002*—were upregulated in L leaves and have been previously implicated in sugar accumulation and carbon partitioning [33,34,35]. Their upregulation is consistent with a putative carbon stress response in the L clone, whereby a non-significant numerical trend toward higher sucrose levels in source leaves may coincide with transcriptional reprogramming that could modify carbon-related metabolic flow. This pattern aligns with a scenario in which clone L displays relatively enhanced expression of defence- and stress-associated transcriptional programmes, which may be inversely associated with rubber biosynthesis. Nevertheless, these observations derive solely from correlative multi-omics data and do not demonstrate causal regulatory relationships.

In addition, genes in these modules were enriched in thiamine metabolism. Thiamine participates in carbon metabolism and plant stress responses [36], and altered thiamine availability can reshape carbon allocation. This enrichment is consistent with a potential compensatory mechanism, whereby the L clone may regulate thiamine-related pathways to buffer carbon metabolism imbalances; however, this hypothesis requires further validation in subsequent experiments.

It is also worth noting that the M clone contained the highest sucrose concentration in latex but failed to achieve high yield performance. This observation aligns with our hypothesis that high sucrose substrate abundance alone is insufficient to drive high rubber biosynthesis, and that transcriptional and metabolic signatures of sucrose breakdown and hexose turnover may be associated with latex yield variation.

Although this study provides integrated transcriptomic and metabolomic evidence linking sucrose allocation patterns to rubber latex yield, a deeper understanding of the underlying mechanisms will require sampling across multiple tapping time points and larger sample sets, alongside direct measurements of photosynthetic parameters and functional validation of candidate enzyme genes. Furthermore, because only one clone was examined for each observed yield rank, the observed differences may be genotype-specific. The genes identified here derive from exploratory correlative analyses, and their specific functions require further validation. Additionally, analyte-specific recoveries varied widely across quantifiable sugars (range: 62–126%), and formal matrix-effect evaluation was not performed owing to the complexity of the plant matrix. Absolute sugar concentrations (mg/g) should therefore be interpreted cautiously, with this analytical limitation considered.

## 4. Materials and Methods

### 4.1. Plant Materials and Experimental Design

Three *H. brasiliensis* clones with distinct genetic background and rubber latex yield (labelled H, M and L) were selected for this study. These clones were identified from 80 *H. brasiliensis* accessions maintained at the Jinghong Rubber Tree Germplasm Resource Garden of the Ministry of Agriculture, China. Latex yield was measured in 2022. One-way ANOVA followed by Tukey’s HSD multiple comparison test (*p* < 0.05) was performed to compare yield performance among the accessions. Three clones with statistically distinct latex yields were selected and designated H, M, and L according to their relative yield ranks. The yield values and statistical comparisons for these clones are summarized in Supplementary Table S1. All trees were transplanted in 2008. Tapping was initiated in early August 2022 using the s/4d3 tapping protocol and continued for three consecutive months. Latex yield was measured with a graduated cylinder at the end of August, September, and October 2022. Latex, leaf, and bark tissues were systematically collected from each tree. Three biological replicates were collected for each tissue type of every clone, resulting in a total of 27 samples. All samples were immediately frozen in liquid nitrogen and stored at −80 °C to preserve the nucleic acids and metabolites for further analysis.

### 4.2. RNA Extraction and cDNA Library Construction

Total RNA was extracted using a commercial Plant RNA extraction kit, following the manufacturer’s protocol. RNA integrity was verified with a NanoDrop 2000C (Thermo Fisher Scientific, Wilmington, DE, USA) spectrophotometer. cDNA libraries were constructed and sequenced using Illumina’s procedures, with sequencing performed on the Illumina HiSeqTM 2000 platform (The Beijing Biomarker Biotechnology Co., Ltd. Wuhan, China).

### 4.3. Transcriptome Data Processing and Bioinformatic Analysis

Raw sequencing data were processed with the FASTX Toolkit to remove adapters and low-quality reads. Clean reads were aligned to the *Hevea* reference genome (the assembly number: ASM1045892v1, https://www.ncbi.nlm.nih.gov/data-hub/genome/GCA_010458925.1/ (accessed on 24 October 2025)) using HISAT2 software. Gene expression levels were quantified by calculating FPKM (Fragments Per Kilobase of transcript per Million Mapped reads). Differential expression analysis was performed using the DESeq R package (R version 4.3.2). Genes with *p*-value < 0.05 and |log_2_(fold change)| > 1 were retained as an exploratory candidate set for hypothesis-generating downstream analyses. BH-adjusted *p*-values for all tested genes are provided in Supplementary Data S1. The candidate genes identified here are classified as exploratory results, rather than differentially expressed genes with FDR significance.

Note that KEGG enrichment and WGCNA-DEG intersection analyses were performed on the uncorrected exploratory candidate gene set (raw *p* < 0.05), serving purely for hypothesis generation, whereas FDR-adjusted DEGs represent statistically validated differential expression.

### 4.4. Extraction and Determination of Sugar Metabolites

Plant samples were freeze-dried and ground into a homogeneous powder. Approximately 20 mg of the powder was accurately weighed and mixed with a methanol:isopropanol:water solution (3:3:2, *v*/*v*/*v*), followed by vortexing and ultrasonic treatment. After centrifugation, the supernatant was mixed with [2h6]D-ribose as the internal standard, dried under a nitrogen stream, and then freeze-dried again. Derivatization was performed using methoxyamine hydrochloride in pyridine, followed by treatment with BSTFA to form trimethylsilyl derivatives for GC-MS analysis. Separation and detection of metabolites were carried out using an Agilent 8890 gas chromatograph coupled with a 5977B mass spectrometer, with a temperature program set to ramp up to 320 °C and helium as the carrier gas at a flow rate of 1 mL/min. Sugar detection and quantification were performed using the MetWare analysis platform (http://www.metware.cn/) based on the Agilent 8890-5977B system.

Compound annotation was achieved by matching mass spectra and retention times against the MetWare database (MWDB). Authentic reference standards (listed in Supplementary Data S4) were prepared at 0.001–50 μg/mL, and calibration curves were constructed for each standard. For each sample, measured peak-area ratios were substituted into the corresponding linear equation, and values were converted to absolute metabolite concentrations (mg/g) using the MetWare built-in formula incorporating unit conversion. The final data are expressed as absolute metabolite content in mg/g. A QC sample was inserted after every 10 experimental samples to monitor analytical reproducibility. To evaluate the accuracy of the quantification method, spiked recovery experiments were performed by adding known amounts of authentic standards to the samples. Recoveries varied substantially across individual sugar analytes, ranging from 62% to 126%, and analyte-specific recovery values are provided in Supplementary Data S4. Analytes with recovery values near the lower or upper bounds of this interval may suffer from substantial extraction-related bias. Formal matrix-effect assessment was not performed in this work. Therefore, absolute quantitative values (mg/g) should be interpreted cautiously.

### 4.5. Statistical Analysis of Metabolomic Data

Three biological replicates per clone (H, M, L) were included for each tissue (leaf, bark, latex). Metabolite intensities were log_2_-transformed and normalized prior to statistical testing. Pairwise comparisons between clones (H vs. M, H vs. L, M vs. L) were performed using two-sample *t*-tests, with Benjamini–Hochberg (BH) correction applied to control the false discovery rate. Metabolites satisfying |log_2_ fold change| ≥ 1 (fold change ≥ 2 or ≤0.5) and BH-adjusted *p*-value (FDR) < 0.05 were defined as DAMs. All metabolomic statistical analyses were implemented in R.

### 4.6. Construction of Weighted Co-Expression Network

Gene expression levels were quantified as FPKM. Genes with FPKM < 3 in more than 3 samples were excluded, and sample and gene quality was assessed using goodSamplesGenes(). The remaining genes were log_2_-transformed (log_2_(FPKM + 1)) and quantile-normalized. A signed co-expression network was constructed using the WGCNA package [37], with networkType = “signed”, Pearson correlation (corFnc = “cor”), and TOMType = “signed”. The soft-thresholding power was set to 12, which satisfied scale-free topology with R^2^ > 0.8, as determined by pickSoftThreshold(). Modules were identified with blockwiseModules() using minModuleSize = 30, deepSplit = 2, and mergeCutHeight = 0.25. Module eigengenes (MEs) were calculated as the first principal component of each module’s expression matrix. Correlations between MEs and metabolites were calculated as Pearson correlations. To control for tissue identity and clone identity, linear models of the form “sucrose ~ ME + tissue + clone identity” were fitted for each module, and partial correlations between each ME and sucrose content were computed after regressing out tissue and clone identity using the ppcor R package. All analyses were performed in R.

### 4.7. qRT-PCR Validation of RNA-Seq Data

qRT-PCR was carried out to validate the RNA-Seq transcriptomic data on three sugar metabolism-associated candidate genes. Three independent biological replicates (*n* = 3) from each clone–tissue combination were used for qRT-PCR. Three technical replicates were performed for each biological sample. The technical replicates were averaged prior to statistical analysis and were not treated as independent observations. *UBC2a* served as the endogenous reference gene in this assay [63]. All primers were designed with Primer Premier 6.0 [64], including gene23505 (F: 5′-GATGAAGGAAGGGAAGAATGCT-3′; R: 5′-GCCATAAGAGTTTGTTTGCGATGG-3′), gene16100 (F: 5′-AATGCAAACAGCTTGGAAGGC-3′; R: 5′-TTTGGTCCTCACCGTTTCTGT-3′), and gene22397 (F: 5′-AATGGGCAGGAATGTTGTTTG-3′; R: 5′-GCTGTAGGGCTCCGATTTC-3′). The amplification efficiencies of all primer pairs were confirmed to be between 90% and 110% using a standard curve method.

RNA templates applied in qRT-PCR were the residual aliquots of total RNA previously extracted for RNA-Seq library construction. First-strand cDNA preparation was conducted according to the procedures described by Mao et al. [65]. The qRT-PCR amplification was performed using the following conditions: initial denaturation at 95 °C for 180 s; 40 cycles of 95 °C for 10 s and 60 °C for 30 s. Relative transcript abundance was quantified by the 2^−ΔΔCT^ approach [66]. IBM SPSS Statistics 26 (Armonk, NY, USA) was used to complete all statistical calculations; one-way analysis of variance (ANOVA) was used to assess statistical significance based on the biological replicates, followed by Duncan’s new multiple range test (*p* < 0.05).

## 5. Conclusions

This study integrates transcriptomics and targeted sugar metabolomics to characterize genotypic differences in source and sink sucrose profiles across three rubber tree clones differing in measured latex yield. Our multi-omics data reveal that these clones harbour distinct sugar metabolite profiles and gene expression signatures linked to sucrose metabolism. Clone L showed numerically higher leaf sucrose concentrations, representing a non-significant numerical trend across the three clones. Clone H exhibited elevated transcript levels of invertase and sucrose synthase genes, and these combined signatures are suggestive of genotypic divergence in sucrose hydrolysis and processing. These correlative observations provide testable hypotheses and candidate molecular resources for high-yield rubber tree breeding. The causal mechanisms and gene functions driving yield variation remain to be validated through subsequent functional experiments.

## Data Availability

The raw sequencing data generated in this study are publicly available at NCBI BioProject (accession PRJNA1025102). All other data supporting the findings of this study are available within the article and its Supplementary Materials.

## Supplementary Materials

The following supporting information can be downloaded at: https://www.mdpi.com/article/10.3390/plants15213442/s1, Data S1: Specifically: Complete differential gene screening results; Data S2: Complete KEGG enrichment results; Data S3: The metabolite matrix and absolute contents; Data S4: The processed GC-MS data, calibration curves, LOD, and LOQ; Data S5: The raw qRT-PCR Ct values; Data S6: WGCNA module memberships, module eigengenes, and module–trait correlations; Table S1: the sample information used in the experiment; Table S2: Tissue-stratified and partial correlation analyses between module eigengenes (MEpink, MEtan) and sucrose content; Figure S1: Coefficient of variation (CV) cumulative distribution of metabolite peaks.
