# Supplementary material for: Sucrose-Related Transcriptomic and Metabolic Profiles Are Associated with Latex Yield Variation in Three Hevea brasiliensis Clones
Source: Plants (Basel). 2026 Nov 5;15(21):3442. doi: 10.3390/plants15213442 (PMC13765739; doi:10.3390/plants15213442)
Supplement: Supplementary file 1 [file plants-15-03442-s001.zip › Supplementary Figure S1.pdf]

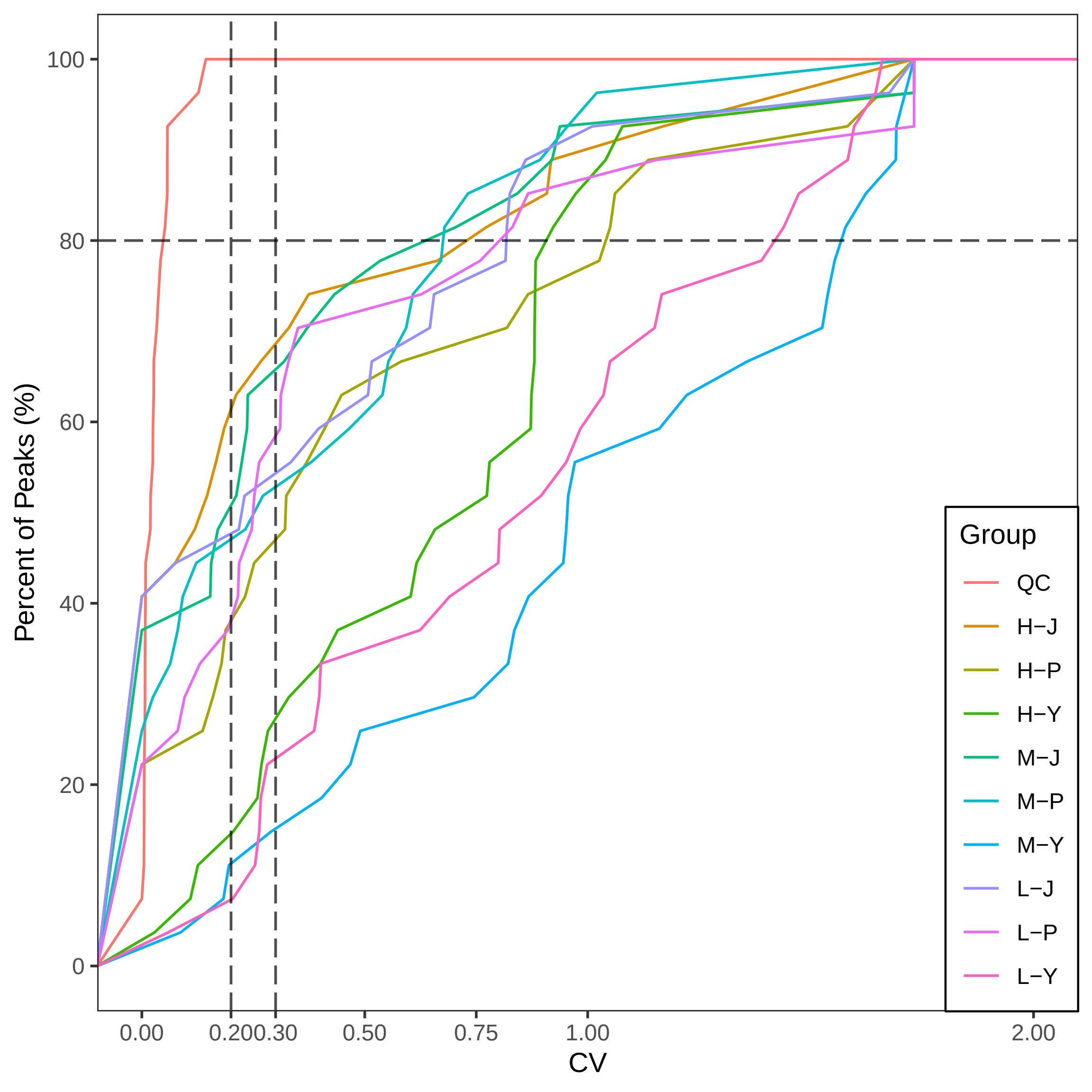

100
80
60
40
20
0
Percent of Peaks (%)
0.00
0.20
0.30
0.50
0.75
1.00
2.00
CV
Group
QC
H−J
H−P
H−Y
M−J
M−P
M−Y
L−J
L−P
L−Y
