# Supplementary material for: Sucrose-Related Transcriptomic and Metabolic Profiles Are Associated with Latex Yield Variation in Three Hevea brasiliensis Clones
Source: Plants (Basel). 2026 Nov 5;15(21):3442. doi: 10.3390/plants15213442 (PMC13765739; doi:10.3390/plants15213442)
Supplement: Supplementary file 1 [file plants-15-03442-s001.zip › Supplementary Table S1.pdf]

Table S1. the sample information used in the experiment.

Variety	Sampling tissue	Sample name	Clone name	Latex yield(ml)		
				2022.08.27	2022.09.24	2022.10.24
High yield rubber	latex	H-latex	Yunyan72-729	650a	800a	600a
High yield rubber	bark	H-bark				
High yield rubber	leaf	H-leaf				
Middle yield rubber	latex	M-latex	Yunyan98-605	150b	200b	200b
Middle yield rubber	bark	M-bark				
Middle yield rubber	leaf	M-leaf				
Low yield rubber	latex	L-latex	Yunyan99-935	50c	50c	30c
Low yield rubber	bark	L-bark				
Low yield rubber	leaf	L-leaf				

Note: The latex yield data in the table above were obtained from tapping initiated in August 2022 using the S/4 d3 tapping protocol, with latex yield measured at the end of August, at the end of September, and at the end of October. The letters following the latex production data indicate statistical significance.
