# Supplementary material for: Sucrose-Related Transcriptomic and Metabolic Profiles Are Associated with Latex Yield Variation in Three Hevea brasiliensis Clones
Source: Plants (Basel). 2026 Nov 5;15(21):3442. doi: 10.3390/plants15213442 (PMC13765739; doi:10.3390/plants15213442)
Supplement: Supplementary file 1 [file plants-15-03442-s001.zip › Supplementary Table S2.pdf]

Supplementary Table S2: Tissue-stratified and partial correlation analyses between module eigengenes (MEpink, MEtan) and sucrose content.

Tissue	Module	r	p	n	p_BH
Leaf	MEpink	0.8231	0.0064	9	0.0385
Leaf	MEtan	0.6246	0.0721	9	0.2164
Bark	MEpink	0.2147	0.5790	9	0.8339
Bark	MEtan	-0.0820	0.8339	9	0.8339
Latex	MEpink	0.1661	0.6694	9	0.8339
Latex	MEtan	0.0935	0.8109	9	0.8339
All (control Tissue+Group)	MEpink	0.7590	0.0000	27	—
All (control Tissue+Group)	MEtan	0.5910	0.0030	27	—

Note: —, not applicable. The partial correlation analysis controls for tissue and yield group within a single model, so BH adjustment is not applicable.
